# The transcriptional repressor Sum1p counteracts Sir2p in regulation of the actin cytoskeleton, mitochondrial quality control and replicative lifespan in *Saccharomyces cerevisiae*

**DOI:** 10.15698/mic2016.02.478

**Published:** 2016-01-18

**Authors:** Ryo Higuchi-Sanabria, Jason D. Vevea, Joseph K. Charalel, Maria L. Sapar, Liza A. Pon

**Affiliations:** 1 Department of Pathology and Cell Biology, Columbia University, New York, NY, USA.; 2 Herbert Irving Comprehensive Cancer Center, Columbia University, New York, NY, USA.; 3 Current address: Department of Neuroscience, University of Wisconsin, Madison, WI, USA.; 4 Current address: Department of Genetics, Stanford University, Stanford, CA, USA.; 5 Department of Biological Sciences, Hunter College and The Graduate Center Biochemistry, Biology and Biopsychology and Behavioral Neuroscience Programs, CUNY, New York, NY 10065, USA. Current address: Weill Institute for Cell and Molecular Biology, Cornell University, Ithaca, NY, USA.

**Keywords:** mitochondria, aging, actin cytoskeleton, sirtuin

## Abstract

Increasing the stability or dynamics of the actin cytoskeleton can extend lifespan in *C. elegans* and *S. cerevisiae*. Actin cables of budding yeast, bundles of actin filaments that mediate cargo transport, affect lifespan control through effects on mitochondrial quality control. Sir2p, the founding member of the Sirtuin family of lifespan regulators, also affects actin cable dynamics, assembly, and function in mitochondrial quality control. Here, we obtained evidence for novel interactions between Sir2p and Sum1p, a transcriptional repressor that was originally identified through mutations that genetically suppress *sir2*∆ phenotypes unrelated to lifespan. We find that deletion of *SUM1* in wild-type cells results in increased mitochondrial function and actin cable abundance. Furthermore, deletion of *SUM1 *suppresses defects in actin cables and mitochondria of *sir2*∆ yeast, and extends the replicative lifespan and cellular health span of *sir2*∆ cells. Thus, Sum1p suppresses Sir2p function in control of specific aging determinants and lifespan in budding yeast.

## INTRODUCTION

Mitochondria have emerged as central regulators of cellular fitness and aging through their functions in central metabolism and reactive oxygen species formation 1,2,3,4,5,6,7. Our previous studies revealed that mitochondria are asymmetrically inherited during yeast cell division, and that this process affects mother and daughter cell fitness and lifespan 8. Asymmetric inheritance of mitochondria also occurs in mammalian cells, and affects cell fate. Specifically, young and old mitochondria are segregated during division in human mammary stem-like cells, and the cell that receives young, presumably fitter mitochondria retains stem cell features while the cell that receives older, presumably less fit mitochondria, differentiate 9. Asymmetric cell division has also been identified in neuronal stem cells, where a diffusion barrier in the endoplasmic reticulum ensures that damaged, dysfunctional proteins characterized by higher ubiquitination profiles are sequestered in differentiating cells 10.

The mechanism underlying asymmetric inheritance of mitochondria in mammalian cells is not well understood. However, one mechanism for inheritance of fitter mitochondria by yeast daughter cells is dependent upon the actin cytoskeleton. Actin cables are bundles of F-actin that align along the mother-bud axis and are essential for trafficking of cargo including mitochondria during cell division. In contrast to many cytoskeleton tracks, which are not motile, actin cables move from buds towards mother cells 11,12. As a result of this retrograde actin cable flow (RACF), mitochondria that use actin cables as tracks for movement from mother cells to buds are effectively “swimming upstream” against the force of RACF, and only fitter mitochondria, that are more motile, are more reduced, and contain less ROS, can move from mother cell to bud and be retained there 8.

There are other lines of evidence suggesting the importance of actin cytoskeletal dynamics in cellular health. For example, aged muscle cells have defects in actin organization in *C. elegans *13. Similarly, loss of actin regulation prevents activation of aged T cells 14. Indeed, recent studies indicate that stabilization of the actin cytoskeleton in *C. elegans *extends lifespan 15. Thus, actin function declines with age in model organisms and mammals, and promoting the function of the actin cytoskeleton can extend lifespan in yeast and *C. elegans*.

Here, we describe a novel interaction between Sir2p and Sum1p in regulation of the actin cytoskeleton, actin-mediated mitochondrial quality control, and lifespan regulation. Silent Information Regulator 2 (*SIR2), *originally described as a regulator of transcriptional silencing 16,17, was the first sirtuin to be described as a lifespan regulator 18. While the link between sirtuins and lifespan regulation is still controversial 19,20,21,22, sirtuin activity has been shown by numerous groups to be involved in mitochondrial quality control in mammals 23,24,25. Moreover, recent work in *S. cerevisiae* indicates that deletion of *SIR2* results in loss of actin polarity, decreased actin cable abundance, decreased rates of RACF, and misfolding of actin protein 8,26,27. This loss of cytoskeletal control results in decreased mitochondrial quality and cellular health span 8. However, it is still unclear how sirtuins exert their role in mitochondrial quality control in mammals.

Sum1p is a promoter-specific repressor, which functions by recruitment of the histone deacetylase, Hst1p 28. Sum1p was originally identified as a suppressor for Sir2p. Since *SIR2* was known as *MAR1* at the time, the gene was named Suppressor of *MAR1*. More specifically, a *sum1-1* mutant was originally characterized as a recessive mutation that ameliorates the defects in gene silencing and mating found in *sir2*∆ cells 29. Sum1p mediates transcriptional silencing of NAD+ biosynthesis genes 30, middle sporulation genes 31, and HM mating-type loci 32. Here, we report that Sum1p and Sir2p inversely regulate actin and mitochondrial maintenance, as well as lifespan.

## RESULTS

### *sum1*∆ cells have increased transcriptional expression of widespread genes

To further characterize the role of Sum1p in transcriptional repression, we used next-generation RNA sequencing (RNAseq) to measure differences in gene expression in *sum1*∆ cells compared to wild-type cells. Predictably, given the role of Sum1p as a transcriptional repressor, we find that a majority of the genes with differential expression (using a P-value < 0.05 as a cutoff) are up-regulated in *sum1*∆ cells: 191 genes have significantly increased expression in *sum1*∆ cells compared to only 23 genes showing decreased expression (Table S1-S2). To identify potential mechanistic pathways that Sum1p may be involved in, we used the FunSpec algorithm 33 to group gene ontology (GO) terms related to the 191 up-regulated genes found in *sum1*∆ cells, and used the REVIGO algorithm to remove redundant GO terms and group related GO terms in semantic similarity-based scatterplots (Fig. S1) 34. In accordance with previous reports, we detect up-regulation of genes involved in reproductive processes including spore formation in *sum1*∆ cells. In addition, the *BNA* family of genes (*BNA1-6*), previously identified in microarray studies, is up-regulated in *sum1*∆ cells. Since these genes are directly involved in de-novo NAD+ biosynthesis, it is possible that deletion of *SUM1* results in increased total cellular NAD+ and positively influences Sir2p activity 30.

### Deletion of *SUM1* increases actin cable abundance in wild-type cells and rescues defects in actin cable abundance found in *sir2*∆ cells

**Figure 1:**
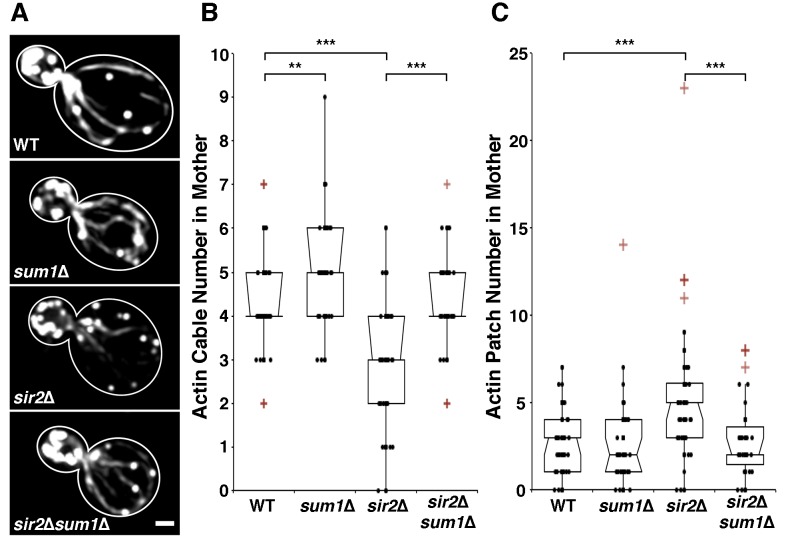
FIGURE 1: Deletion of *SUM1* suppresses defects in cytoskeletal integrity of *sir2*∆ cells. **(A)** Wild-type, *sum1*∆, *sir2*∆, and *sir2*∆ *sum1*∆ cells were stained with rhodamine-phalloidin as described in Materials and Methods. Scale bar represents 1 µm. Outlines were drawn from brightfield images. **(B)** Notched dot box plot of the number of polarized actin cables greater than half the length of the mother cell. The central band in the box represents the median, boxes indicate the middle quartiles, whiskers extend to the 5^th^ and 95^th^ percentiles, and red points indicate outliers (defined as quartile ± 1.5x the interquartile range). n = 45 - 62 cells per strain. Data is representative of 3 independent trials. ** = P < 0.01, *** = P < 0.001. P values were calculated using Kruskal-Wallis testing. **(C)** Notched dot box plot of the number of actin patches in the mother cell. Here, actin patches in the mother cell were quantified by visual inspection of cells bearing a small to medium bud (defined as cells with a ratio of bud:mother diameter of 0.2 to 0.6). During polarized growth, endocytosis and formation of actin patches occurs primarily in daughter cell. Therefore, mislocalization of patches into the mother cell early in the cell cycle is used as a marker for loss of polarity. n = 45 - 62 cells per strain. Data is representative of 3 independent trials. *** = P < 0.001. P values were calculated using Kruskal-Wallis testing.

**Figure 2:**
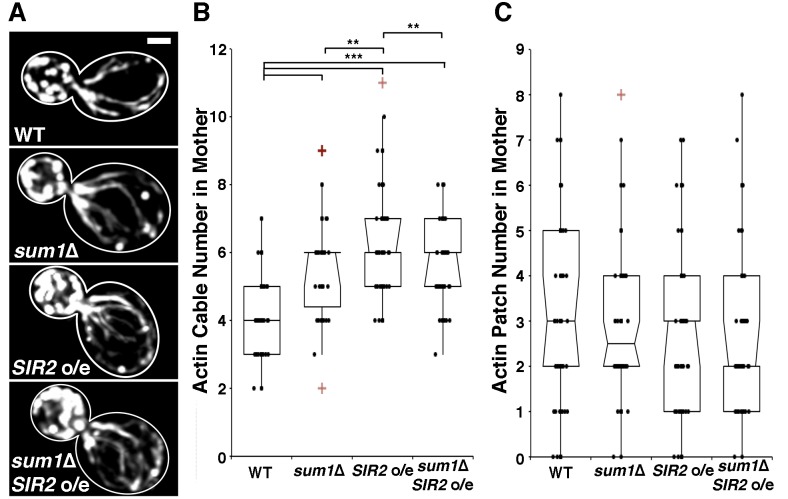
FIGURE 2: Overexpression of *SIR2* and deletion of *SUM1* have complementary roles in regulation of actin cytoskeletal integrity. **(A)** Wild-type, *sum1*∆, *SIR2 *overexpressing (*SIR2* o/e), and *sum1*∆ *SIR2* o/e cells were stained with rhodamine-phalloidin as described in Materials and Methods. Scale bar represents 1 µm. Outlines were drawn from brightfield images. **(B)** Notched dot box plot of the number of actin cables in wild-type, *sum1*∆, *SIR2 *o/e, and *sum1*∆ *SIR2* o/e cells. **(C)** Number of actin patches in the mother cell of wild-type, *sum1*∆, *SIR2 *o/e, and *sum1*∆ *SIR2* o/e cells. n = 56 - 69 cells per strain. Data is representative of 2 independent trials. ** = P < 0.01, *** = P < 0.001. P values were calculated using Kruskal-Wallis testing.

Deletion of *SIR2* results in depolarization of the actin cytoskeleton, a significant decrease in actin cable number and rate of RACF, and defects in actin protein folding 8,27. We confirmed the decline in actin cable abundance in *sir2*∆ cells (Fig. 1A-B). Conversely, overexpression of *SIR2* has the opposite effect (Fig. 2A-B). Furthermore, we find that deletion of *SUM1* results in an increase in actin cable abundance to levels higher than those observed in wild-type cells (Fig. 1A-B) and an increase in the rate of RACF (Fig. S2).

Since deletion of *SUM1* results in an increase in NAD+ biosynthetic enzymes, which in turn may increase Sir2p activity, it is possible that Sum1p function in actin cytoskeletal integrity may be due to effects on Sir2p. Indeed, while overexpression of *SIR2 *results in an increase in actin cable abundance, deletion of *SUM1* in *SIR2*-overexpressing cells does not have an additive effect on actin cable abundance (Fig. 2A-B). These findings indicate that Sum1p and Sir2p antagonistically regulate the actin cytoskeleton through similar mechanisms. On the other hand, we find that deletion of *SUM1* ameliorates the defects in actin cable abundance found in *sir2*∆ cells (Fig. 1A-B). Thus, Sum1p has additional functions beyond direct regulation of Sir2p activity.

To investigate the role of Sum1p in the polarization of the actin cytoskeleton, we examined the distribution of actin patches, which are endocytic vesicles coated with F-actin that are primarily found in the bud during polarized yeast cell division. Localization of 4 or more actin patches in mother cells, using conventional fluorescence imaging, is indicative of loss of polarity in the actin cytoskeleton 35. Deletion of *SIR2* results in an elevated number of actin patches in the mother cell. This polarity defect in *sir2*∆ cells, like the defect in actin cable abundance, is ameliorated by the deletion of *SUM1 *(Fig. 1C). We find no major differences in actin patch localization in wild-type or *sum1*∆ cells overexpressing *SIR2 *(Fig. 2C).

### Deletion of *SUM1* promotes mitochondrial quality in wild-type cells and rescues mitochondrial function in *sir2*∆ cells

Recent work from our laboratory identified a novel role for Sir2p in regulation of RACF 8 and in regulating mitochondrial quality control, in part, through its effects on RACF. Since deletion of *SUM1* also results in an increase in RACF (Fig. S2), we studied the effect of deletion of *SUM1* on mitochondrial function by analysis of two parameters: 1) mitochondrial redox state and 2) mitochondrial motility. Mitochondrial redox state was measured using mitochondria-targeted redox-sensing GFP1 (mito-roGFP1), which contains two surface-exposed cysteines. Oxidation or reduction of the cysteines occurs in response to the redox state of the environment and alters the excitation spectrum of roGFP1 36. When roGFP1 is targeted to yeast mitochondria, its fluorescence ratio serves as an effective biosensor for mitochondrial redox state 37.

**Figure 3:**
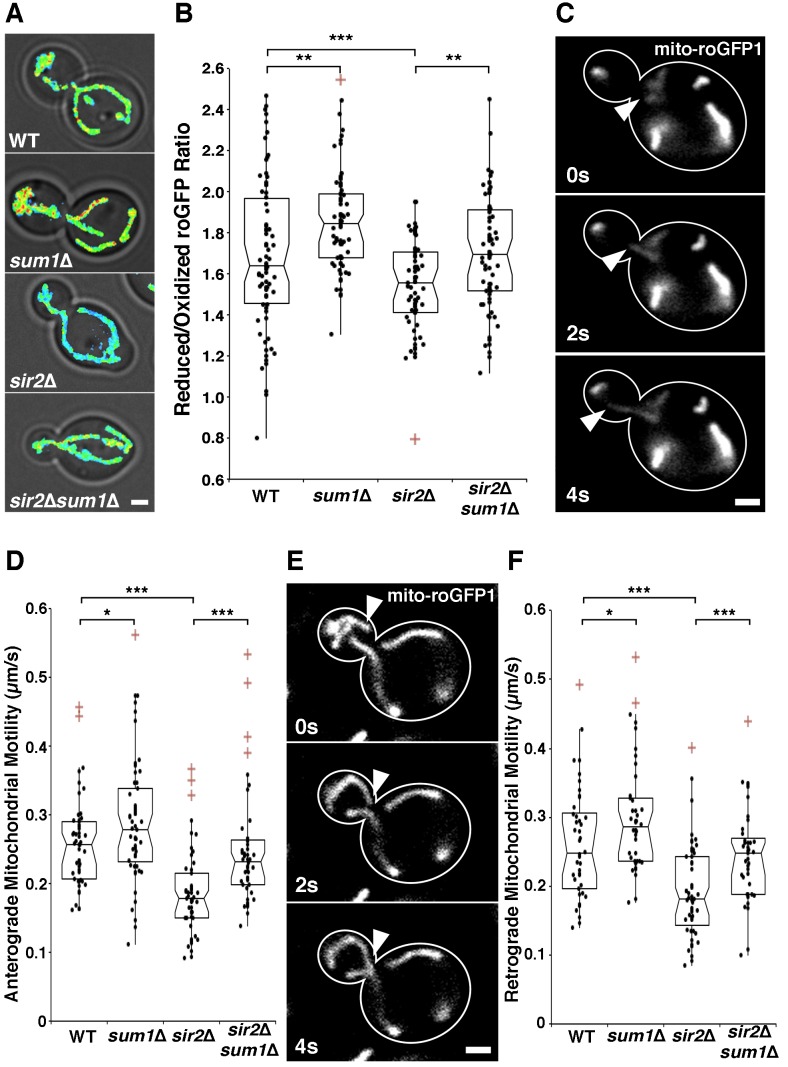
FIGURE 3: Deletion of *SUM1* ameliorates defects in mitochondrial function in *sir2*∆ cells. **(A)** mito-roGFP1 was used to visualize redox state of mitochondria in wild-type, *sum1*∆, *sir2*∆, and *sir2*∆ *sum1*∆ cells. Images are reduced:oxidized mito-roGFP1 ratios overlaid on phase images. Higher numbers and warmer colors indicate more reducing mitochondria. Scale bar, 1 µm. **(B)** Notched dot box plot of the average reduced:oxidized mito-roGFP1 ratio in wild-type, *sum1*∆, *sir2*∆, and *sir2*∆ *sum1*∆ cells. n = 53 - 77 cells per strain. Data is representative of 3 independent trials. **(C)** Time-lapse frames showing the tip of a Cit1p-GFP-labelled mitochondrial tubule undergoing anterograde movement. **(D)** Notched dot box plot of anterograde mitochondrial velocity in wild-type, *sum1*∆, *sir2*∆, and *sir2*∆ *sum1*∆ cells. n = 48 - 54 cells per strain. Data is pooled from 3 independent trials. **(E)** Time-lapse frames showing the tip of a mito-roGFP1-labelled mitochondrial tubule undergoing retrograde movement. **(F)** Notched dot box plot of retrograde mitochondrial movement in wild-type, *sum1*∆, *sir2*∆, and *sir2*∆ *sum1*∆ cells. n = 48 - 54 cells per strain. Data is pooled from 3 independent trials. * = P < 0.05, ** = P < 0.01, *** = P < 0.001. P values were calculated using Kruskal-Wallis testing. Bars: 1μm. Cell outlines are shown in white.

**Figure 4:**
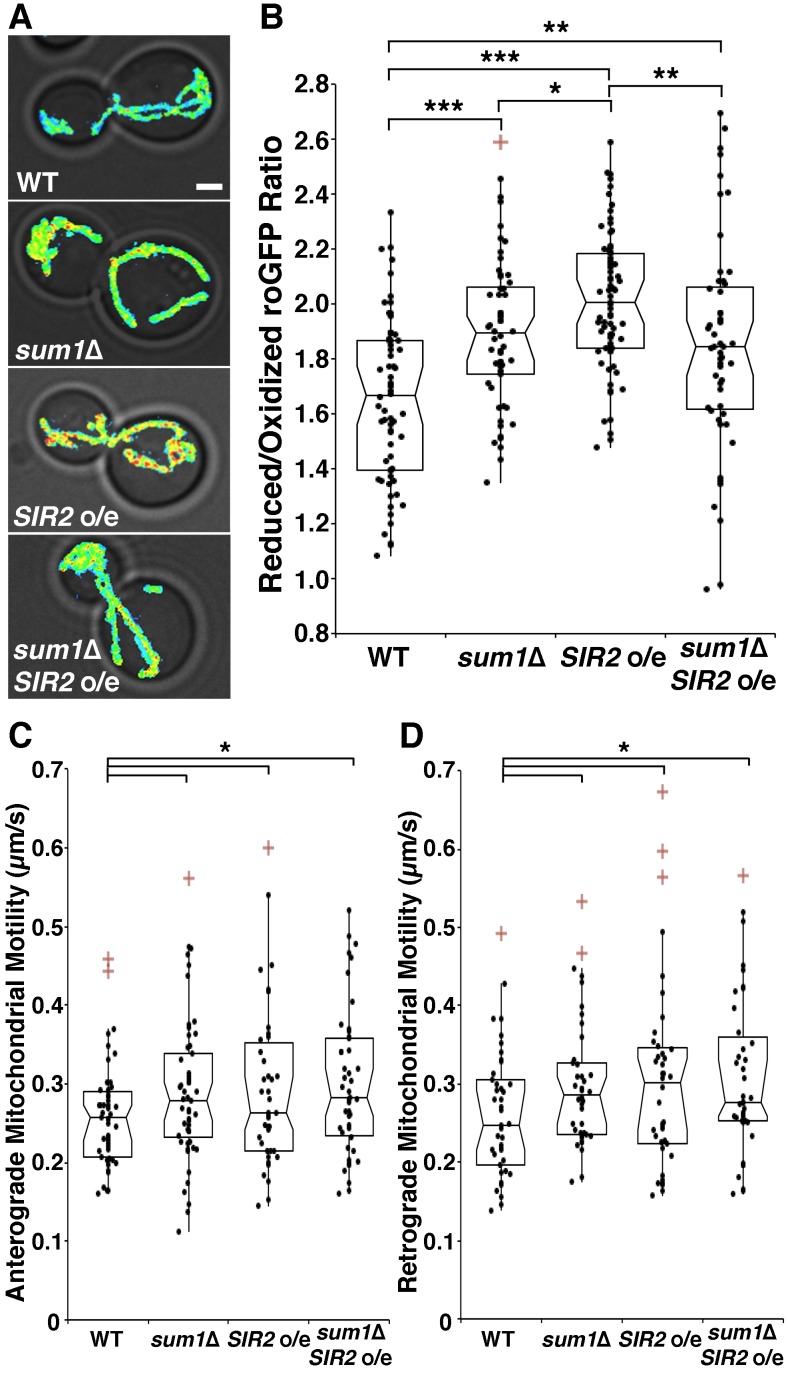
FIGURE 4: Overexpression of *SIR2* and deletion of *SUM1* have complementary roles in regulation of mitochondrial quality and function. **(A)** Reduced:oxidized mito-roGFP1 ratios overlaid on brightfield images for wild-type, *sum1*∆, *SIR2 *o/e, and *sum1*∆ *SIR2* o/e cells. Higher numbers and warmer colors indicate more reducing mitochondria. Scale bar, 1 µm. **(B)** Notched dot box plot of the average reduced:oxidized mito-roGFP1 ratio in wild-type, *sum1*∆, *SIR2 *o/e, and *sum1*∆ *SIR2* o/e cells. n = 53 - 66 cells per strain. Data is representative of 3 independent trials. **(C-D)** Notched dot box plot of anterograde and retrograde mitochondrial movements for wild-type, *sum1*∆, *SIR2 *o/e, and *sum1*∆ *SIR2* o/e cells. n = 43 - 54 cells per strain. Data is pooled form 3 independent trials. * = P < 0.05, ** = P < 0.01, *** = P < 0.001. P values were calculated using Kruskal-Wallis testing. Bars: 1 μm. Cell outlines are shown in white.

Consistent with previous findings, *sir2*∆ cells have a significant decline in mitochondrial function as measured by more oxidizing environments (Fig. 3A-B) and decreased anterograde (Fig. 3C-D) and retrograde mitochondrial velocities (Fig. 3E-F). Overexpression of *SIR2* has the opposite effect (Fig. 4). More importantly, we find that deletion of *SUM1* in wild-type cells results in a modest, but significant improvement in mitochondrial redox state and motility. Additionally, we find that deletion of *SUM1* in *sir2*∆ cells results in complete rescue of mitochondrial redox state and mitochondrial motility to wild-type levels. These data provide further evidence of a link between cytoskeletal integrity and mitochondrial function and that *SUM1* and *SIR2* act antagonistically in these processes.

Consistent with our findings on the actin cytoskeleton, we find that deletion of *SUM1* does not further enhance mitochondrial quality in cells overexpressing *SIR2 *(Fig. 4A-B). The lack of an additive effect on mitochondrial fitness provides further evidence that Sir2p and Sum1p function in similar mechanistic pathways to control the actin cytoskeleton and mitochondria.

### Deletion of *SUM1* promotes lifespan in *sir2*∆ cells

**Figure 5:**
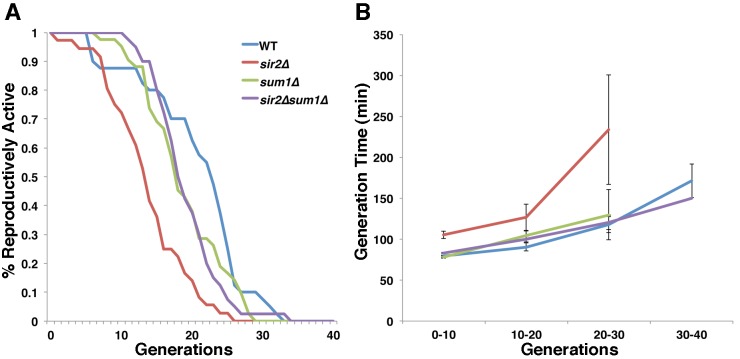
FIGURE 5: Deletion of *SUM1* rescues defects in RLS and cellular health span of *sir2*∆ cells. **(A)** RLSs of wild-type, *sir2*∆, *sum1*∆, and *sir2*∆*sum1*∆ cells were determined as described in Materials and Methods. **(B)** Mean generation time was determined during the RLS assay as described in Materials and Methods. Error bars represent SEM. n = 40 cells per strain. Data is representative of 2 independent trials.

We next investigated whether the Sum1p-mediated increase in mitochondrial quality is physiologically relevant to cellular health and lifespan. Two distinct forms of cellular aging are studied in yeast. Chronological lifespan, the survival time of non-dividing yeast in a saturated culture, is a model for stress resistance in post-mitotic cells. Replicative lifespan (RLS) is a model for aging of division-competent cells and is measured as the number of times a cell can divide prior to senescence. We find that the mean RLS of wild-type, *sir2*∆, *sum1*∆, and *sir2*∆* sum1*∆ are 21.4 ± 2.7, 13.7 ± 0.95, 19.0 ± 0.90 and 19.3 ± 0.70 generations, respectively. Thus, we find that deletion of *SUM1* does not have a significant effect on RLS. More importantly, we find that the decrease in RLS produced by deletion of *SIR2* is suppressed by deletion of *SUM1* (Fig. 5A).

Cellular health in yeast is measured by mean generation time, which increases as the cell ages 38. Deletion of *SUM1* does not affect health span: the mean generation time of *sum1*∆ cells is indistinguishable from that observed in wild-type cells. We confirmed that deletion of *SIR2* results in a significant increase in generation time, even at a young age (Fig. 5B). Moreover, we find that the decreased health span produced by deletion of *SIR2* is ameliorated by deletion of *SUM1*. Thus, deletion of *SUM1* restores lifespan and health span in *sir2*∆ cells.

## DISCUSSION 

Previous studies revealed that deletion of *SIR2* results in defects in folding of the actin protein, as well as a decrease in actin cable abundance and dynamics 8,27, which in turn results in inheritance of less fit mitochondria by yeast daughter cells and reduced cellular health span 8. Other studies showed that the *sum1-1 *mutation, which affects transcriptional regulation by Sum1p, rescues mating defects in *sir2*∆ cells 28,29,32. Here, we show that *SUM1 *acts to antagonize multiple *SIR2* functions. In particular, deletion of *SUM1* ameliorates defects in actin cytoskeletal integrity, mitochondrial quality, and lifespan characteristic of *sir2*∆ cells.

We find that deletion of *SUM1* alone results in an increase in actin cable abundance and RACF velocity and in improved mitochondrial function. Furthermore, deletion of *SUM1* in *sir2*∆ cells completely rescues defects in the actin cytoskeleton and mitochondria that occur in *sir2*∆ cells. To determine the relationship between the Sum1p and Sir2p pathways, we deleted *SUM1* in *SIR2*-overexpressing cells, which display an increase in actin cable abundance and mitochondrial function 8 similar to that found in *sum1*∆ cells. We find that overexpression of *SIR2* and deletion of *SUM1* do not have an additive effect on actin cable abundance or mitochondrial function. This is not due to an independent cellular limit on cable abundance or mitochondrial function. Overexpression of Tpm1p, a tropomyosin isoform that stabilizes actin cables, results in a higher number of actin cables compared to that observed in *sum1*∆, *SIR2*-overexpressing yeast. Similarly, mitochondria are more reduced in yeast undergoing respiration-driven growth than in *sum1*∆, *SIR2*-overexpressing yeast (data not shown). Thus, our findings indicate that Sum1p and Sir2p affect the actin cytoskeleton and mitochondrial function through the same pathway.

Consistent with this, we find that deletion of *SUM1* rescues defects in RLS and cellular health span seen in *sir2*∆ cells. That is, the RLS of *sir2*∆ *sum1*∆ yeast is equal to that of *sum1*∆ cells. Interestingly, *sum1*∆ cells do not have increased RLS compared to wild-type cells, despite having increased actin cable count and higher mitochondrial quality. This may be due to the pleiotropic effects of *SUM1* deletion. Indeed, previous studies have reported a role for Sum1p in regulation of the microtubule cytoskeleton 39,40. It is possible that loss of microtubule maintenance or some other unidentified function of Sum1p – independent of Sir2p activity – contributes to lifespan defects, which mask the positive effects of actin and mitochondrial quality.

Overall, we obtained evidence for a novel role for Sum1p, a suppressor of Sir2p, in regulation of actin cytoskeleton and mitochondrial control. We also identified a novel role for Sum1p in lifespan control and cellular health span. Finally, we obtained evidence that *SUM1* and *SIR2* affect actin, mitochondria, and lifespan through effects on the same pathway.

Previous reports indicate that Sir2p can also affect RLS through effects on generation of extrachromosomal rDNA circles, which are selectively retained in mother cells 18; activation of catalase in daughter cells after cytokinesis 27; and asymmetric inheritance of protein aggregates 41. The actin cytoskeleton, which is required for asymmetric inheritance of mitochondria, catalase activity and protein aggregates, is also affected by both Sum1p and Sir2p. Thus, while it’s clear that increasing RACF results in lifespan extension through effects on inheritance of fitter mitochondria by yeast daughter cells, it is possible that other actin-dependent or actin-independent asymmetric inheritance events contribute to the extended lifespan observed in *sum1*∆ or *SIR2*-overexpressing cells.

Finally, *sum1-1*, the allele that suppresses Sir2p, affects the transcriptional activity of Sum1p 42. Therefore, we favor the notion that *SUM1* controls lifespan through its function as a transcriptional regulator. Our transcriptome analysis confirmed a significant increase in NAD+ biosynthetic enzymes (*BNA1-6*) 30. It is possible that the increased expression of the *BNA* family genes results in an increased available pool of NAD+, which increases Sir2p activity. Since deletion of *SUM1* can also extend lifespan in *sir2*∆ cells, it is possible that elevated NAD+ levels in *sum1*∆ cells extend lifespan through effects on other NAD+-dependent protein deacetylases, such as Hst1 and Hst2. Alternatively, there may be other pathways activated in *sum1*∆ cells that promote longer lifespan. Indeed, we find that sum1∆ cells show greater than 1000-fold overexpression of several previously uncharacterized open reading frames, such as *YGl138c, YLR308W, *and *YNL318c*. It is possible that these genes contribute to Sir2p-dependent and/or Sir2p-independent control of lifespan.

## Materials and Methods

### Yeast Growth Conditions

Yeast cells were cultivated and manipulated as previously described 43 and are derivatives of the BY4741 strain (*MAT***a ***his3*∆*1 leu2*∆*0 met15*∆*0 ura3*∆*0*) from Open Biosystems (Huntsville, AL). All experiments were carried out with cultures grown to mid-log phase (OD_600_ 0.1-0.3) unless otherwise noted. For all imaging studies, synthetic complete (SC) media was used with dropouts where needed. For all non-imaging studies, rich, glucose-based medium (yeast-peptone-dextrose, YPD) was used for strains not requiring selection and SC with corresponding dropouts was used for strains requiring selection.

### Yeast strain construction

Knockout strains **(Table S4) **were created by replacing the gene of interest with *LEU2* or *KanMX6 *cassettes using primers listed in **Supplemental Table 5**. BY4741 strains were transformed using the lithium acetate method with a PCR product containing the coding regions of the selection marker and sequences directly upstream of the start codon and downstream of the stop codon of the gene being knocked out. The *LEU2* auxotrophic selection marker was removed from strain RHY117 (*MATa his3*∆*1 leu2*∆*0 met15*∆*0 ura3*∆*0 SIR2-3HA-kanMX6 sum1*∆*::0 sir2*∆*::KanMX6*) using a galactose-inducible Cre recombinase on plasmid pSH62 (Euroscarf, University of Frankfurt) 44 induced for 4 hrs at 30°C in media containing galactose. Excision of *LEU2* was confirmed by failure to grow on SC-Leu media.

### Visualization of the actin cytoskeleton with rhodamine-phalloidin

Cells were grown to mid-log phase in SC with dropouts where needed and fixed by incubation in 3.7% paraformaldehyde added directly to the growth medium at 30°C with shaking for 50 min. Fixed cells were washed with wash solution (0.025 M KPi pH 7.5, 0.8 M KCl) three times and with PBT (PBS containing 1% w/v BSA, 0.1% v/v Triton X-100, 0.1% w/v sodium azide) once. Actin was stained with 1.65 µM rhodamine-phalloidin in PBT (Molecular Probes, Eugene, OR) for 35 min at RT in the dark. Cells were then washed three times with PBS, resuspended in mounting solution (0.1% w/v p-phenylenediamine and 90% v/v glycerol in PBS) and mounted on microscope slides for visualization.

Fluorescence microscopy was performed on a Zeiss AxioObserver.Z1 microscope (Carl Zeiss Inc., Thornwood, NY) equipped with a metal halide lamp, standard rhodamine filter (Zeiss filter set 43 HE; excitation 550/25, dichroic FT 570, emission 605/70) and an Orca ER cooled CCD camera (Hamamatsu Photonics, Bridgewater, NJ) and driven by Axiovision software (Carl Zeiss Inc., Thornwood, NY). Using a 100x/1.3 NA EC-PlanNeoFluar objective lens, z-series were collected through the entire cell at 0.3 µm intervals using 1x1 binning, 50 ms exposure, and analog gain at 216. Images were deconvolved using a constrained iterative restoration algorithm (Volocity, Perkin-Elmer, Waltham, MA) with the following parameters: 620 nm emission wavelength, 60 iterations, and 100% confidence interval. Cells with small and medium-sized buds (0.20 to 0.60 bud-to-mother diameter ratio) were selected for quantification of actin cables and patches in the mother cell. Actin cable abundance was scored as number of actin cables parallel to the mother-bud axis spanning minimally half of the mother cell diameter.

### Measurement of redox state using mito-roGFP1

mito-roGFP1 was expressed from a centromeric plasmid containing the mitochondrial targeting sequence of ATP9 fused to roGFP1 45. Strains expressing mito-roGFP1 were grown to mid-log phase and 1.5 µL of cell suspension was applied to a microscope slide, covered with a cover slip, and imaged immediately for a maximum of 15 min. mito-roGFP1 was imaged on the Zeiss AxioObserver.Z1 microscope described above using a modified GFP filter (Zeiss filter set 46 HE with excitation filter removed, dichroic FT 515, emission 635/30). Using a 100x/1.3 NA EC-PlanNeoFluar objective lens, z-series were collected through the entire cell at 0.3 µm intervals using 1x1 binning, 365 nm LED at 25% power with 100 ms exposure time for oxidized form and 470 nm LED at 100% power with 100 ms exposure time for reduced form, with analog gain at 216. Images were deconvolved using a constrained iterative restoration algorithm with the following parameters: 507 nm emission wavelength, 60 iterations over 100% confidence interval. For quantification of redox state, Volocity was used to calculate the ratio with background selection and thresholding steps.

### Analysis of velocity of mitochondrial movement

For time-lapse imaging of mitochondria, mito-roGFP1 was excited only at 470 nm in a single plane at the center of the mother cell at 1-sec intervals for a total of 30 sec using 1x1 binning, 75 ms exposure, and analog gain of 216. The change in position of the tip of a mitochondrial tubule was recorded as a function of time. Mitochondrial movement was scored if the tubule tip underwent three or more consecutive movements in the same direction. Image enhancement and analysis were performed using Volocity software.

### Determination of RLS and generation time

RLS measurements were performed on YPD plates at 30°C. Single colonies of each yeast strain were suspended in liquid YPD and grown at 30°C with shaking to mid-log phase. A 2-µL aliquot of cell suspension was applied to a YPD plate. Small-budded cells were isolated and arranged in a matrix using a micromanipulator mounted on a dissecting microscope (Carl Zeiss, Thornwood, NY). After the initial cell division, mother cells were removed and discarded. The remaining daughter cells were named virgin mother cells, and after each successive replication, daughter cells were removed and counted until the virgin mother cell reached senescence. Mean generation time was recorded as the time between successive replications. RLS measurements were done without pause and plates were never moved to 4°C for storage due to a potential cold-sensitive nature of the *sum1*∆ cells 40.

### Analysis of retrograde actin cable flow

Cells expressing Abp140-GFP were grown to mid-log phase in SCRaff media. 1.5 µL of cell suspension was spread over the surface of a glass slide and covered with a cover slip. Slides were imaged within 2 minutes after slide preparation. Abp140-GFP was imaged using a Zeiss 100x/1.4 Plan-Apochromat objective lens, a 470 nm LED at 80% power for excitation, and a standard GFP filter (Zeiss filter set 46 HE; dichroic FT 515, emission 535/30). Images were collected at a focal plane 0.5 - 1 µm above the center of the mother cell, at 0.9-sec intervals for a total of 15 sec using 1x1 binning, 200 ms exposure, and analog gain of 255. Velocity was measured by measuring the change in position of the tip of moving or elongating cables, or the movement of fiduciary marks along actin cables, as a function of time, as previously described 12.

### Other methods

All non-parametric statistical testing and production of box plots were performed using the Analyze-it add-on for Microsoft Excel.

RNA isolation was performed as per manufacturer’s guide using a Qiagen RNeasy Mini Kit (#74104, Qiagen, Germantown, MD). RNA quality was assessed and RNA Integrity Number (RIN) scores were all above 10 using a Plant RNA Nano chip. RNA-seq was performed on an Illumina HiSeq2000 generating 200m 100 bp single end reads per lane, and differential gene expression analysis of wild-type and *sum1*∆ cells was performed by the Columbia Genome Center. Data was organized into gene ontology (GO) terms using the *Saccharomyces *Genome Database’s GO Annotation algorithm. REVIGO was used to remove redundant GO terms and group-related GO terms in semantic similarity-based scatterplots 34.

## SUPPLEMENTAL MATERIAL

Click here for supplemental data file.

All supplemental data for this article are also available online at http://microbialcell.com/researcharticles/the-transcriptional-repressor-sum1p-counteracts-sir2p-in-regulation-of-the-actin-cytoskeleton-mitochondrial-quality-control-and-replicative-lifespan-in-saccharomyces-cerevisiae/.
