# Supplementary material for: The transcriptional repressor Sum1p counteracts Sir2p in regulation of the actin cytoskeleton, mitochondrial quality control and replicative lifespan in Saccharomyces cerevisiae
Source: Microb Cell. 2016 Jan 18;3(2):79–88. doi: 10.15698/mic2016.02.478 (PMC5349106; doi:10.15698/mic2016.02.478)
Supplement: Supplementary file 1 [file mic-03-079-s01.pdf]

Supplemental Data

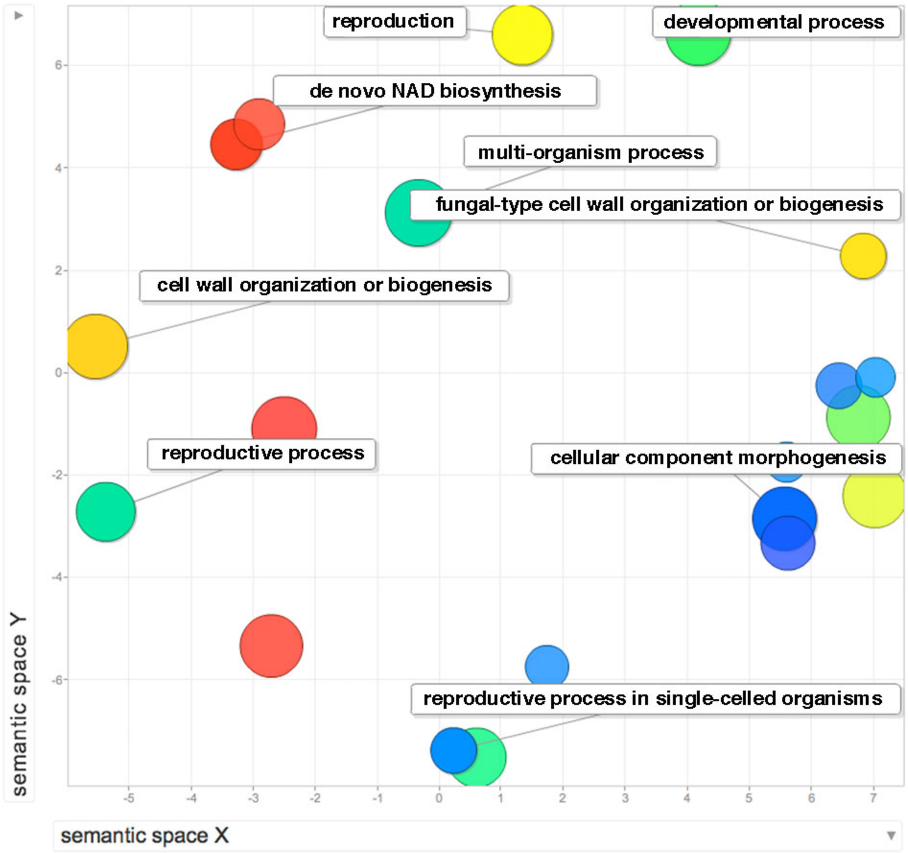

Fig. S1. Comparative transcriptome analysis reveals Sum1p's role in regulation of NAD biosynthesis, cell wall organization, and reproduction. Rna isolation was performed in wild-type and *sum1*Δ cells using a Qiagen RNeasy Mini Kit. RNA-seq was performed as described in *Materials and Methods*, and differential gene expression between wild-type and *sum1*Δ cells were calculated and are available in Supplemental Table 1-2. Data for overexpressed genes were organized into gene ontology (GO) terms using the *Saccharomyces* Genome Database GO Annotation algorithm – all GO terms can be found in Supplemental Table 3. GO terms were further organized through REVIGO to remove redundant GO terms and group related GO terms in a semantic similarity-based scatterplot. Bubble color indicates the uniqueness of each GO term – the similarity of the GO term to other GO terms – where warmer, redder colors indicate higher uniqueness, and cooler, bluer colors indicate lower uniqueness. Bubble size indicates the frequency of the GO term in the underlying database.

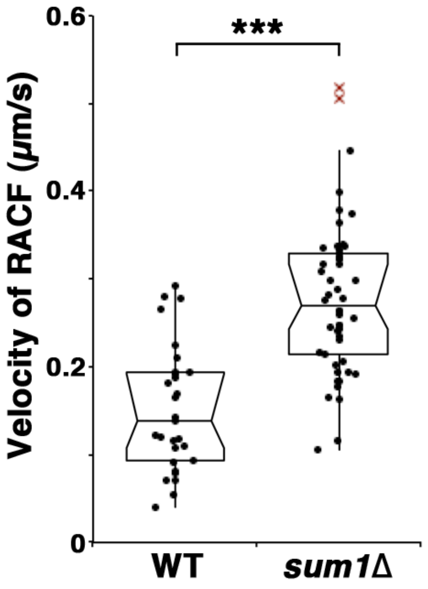

Fig. S2. Deletion of *SUM1* results in increased RACF. Notched-dot box plot of the velocity of RACF in WT and *sum1*Δ cells. Wild-type and *sum1*Δ cells expressing Abp140-GFP were imaged at 0.9-sec intervals for 15 sec as described in *Materials and Methods*. Velocity measurements were determined by tracking movement of cables or fiduciary marks along the actin cables as previously described [12]. *** = $p < 0.001$. p-values were calculated using non-parametric Kruskal-Wallis testing. n = 29 cells for wild-type and 50 cells for *sum1*Δ. Data is pooled from 3 independent trials.

Gene	Gene Name	Fold Change	p-value
YIL130W	ASG1	1.649666981	0.037948076
YBR084W	MIS1	1.652338918	0.048551142
YAR042W	SWH1	1.666670902	0.039089908
YIL117C	PRM5	1.675506991	0.047300478
YGR187C	HGH1	1.703684959	0.045690916
YER115C	SPR6	1.731887406	0.040700141
YMR272C	SCS7	1.731993629	0.031696193
YOL011W	PLB3	1.776705443	0.024283181
YOR011W	AUS1	1.784641816	0.030112853
YPL184C	MRN1	1.796784261	0.028566347
YEL023C	N/A	1.804878299	0.027093525
YHR154W	RTT107	1.805454729	0.027154016
YGR032W	GSC2	1.809879998	0.009953529
YLR084C	RAX2	1.825727357	0.020713733
YPR194C	OPT2	1.837323883	0.023483689
YER060W	FCY21	1.996682056	0.010289448
YDR409W	SIZ1	2.004975139	0.010177886
YHL048W	COS8	2.010414697	0.009944426
YGR258C	RAD2	2.029676316	0.008979972
YDL223C	HBT1	2.034988128	0.008527741
YDR317W	HIM1	2.045496593	0.035935947
YDL167C	NRP1	2.062781017	0.007612058
YDR503C	LPP1	2.068381712	0.008715474
YBR063C	N/A	2.07959056	0.030521144
YLL004W	ORC3	2.091833632	0.006876399
YOR347C	PYK2	2.109725671	0.00545232
YLR042C	N/A	2.125430683	0.014641269
YAR071W	PHO11	2.132023357	0.006567578
YJR107W	N/A	2.163951644	0.004002729
YHL050C	N/A	2.203687776	0.040546033
YLR054C	OSW2	2.217728085	0.006075381
YOR355W	GDS1	2.301476734	0.001416495
YLR233C	EST1	2.304997428	0.00188856
YDL079C	MRK1	2.313730682	0.008629998
YFL014W	HSP12	2.32580821	0.005175414
YFR047C	BNA6	2.428769297	0.001052625
YNL336W	COS1	2.448761574	0.001418506
YLR341W	SPO77	2.459161104	0.01378499
YBR076W	ECM8	2.499509118	0.023551603
YIR030C	DCG1	2.564039523	0.040924568

YDR403W	DIT1	2.598309294	0.023346661
YEL057C	SDD1	2.610922446	0.03944945
YJL089W	SIP4	2.725381781	0.029779034
YMR069W	NAT4	2.768434529	0.019848511
YIR028W	DAL4	2.787343158	0.008344466
YDR541C	N/A	2.795336811	0.001150582
YCL027W	FUS1	2.801962719	0.000243901
YJR027W	N/A	2.866747167	0.001761362
YER084W	N/A	2.915722604	0.028030921
YIL037C	PRM2	2.940608586	0.000414913
YDL186W	N/A	2.943423172	0.012910561
YHL012W	N/A	2.959096699	0.005108058
YDR273W	DON1	3.03151479	0.006275158
YDL035C	GPR1	3.071766796	2.17E-06
YML066C	SMA2	3.081210083	0.001656355
YCR001W	N/A	3.313136514	0.016503259
YBR250W	SPO23	3.48587831	0.005753874
YLR040C	AFB1	3.554415348	9.23E-06
YAR035W	YAT1	3.55443229	0.00050502
YGR087C	PDC6	3.557522867	0.017172214
YLL005C	SPO75	3.567075475	4.40E-06
YNL202W	SPS19	3.638837676	0.01156495
YDR534C	FIT1	3.64725991	3.19E-05
YOR237W	HES1	3.717042843	0.006607194
YIR027C	DAL1	3.796791441	0.000735891
YNL018C	N/A	3.812346913	0.003162094
YER188W	N/A	3.843133923	0.000246116
YAL037C-A	N/A	3.860810675	0.019793693
YHR054C	N/A	3.888258783	0.01667513
YOR071C	NRT1	3.902683177	2.00E-07
YGR144W	THI4	3.903863718	0.006325109
YOR388C	FDH1	3.942262377	0.043350755
YMR017W	SPO20	3.997545554	0.009518141
YPR054W	SMK1	4.131438055	7.13E-07
YOR072W-A	N/A	4.176592659	0.02449823
YFR012W	DCV1	4.183053972	0.001929834
YJL219W	HXT9	4.191142578	0.009124011
YLR049C	N/A	4.216728814	2.07E-07
YLR231C	BNA5	4.25471397	1.55E-07
YJR079W	N/A	4.286502992	0.040536793
YNR064C	N/A	4.308057482	0.000198896

YOR242C	SSP2	4.349664626	0.001083286
YAL067C	SEO1	4.466380868	0.00048827
YOR365C	N/A	4.467126635	2.36E-06
YFR012W-A	N/A	4.489529759	0.018507919
YGL258W-A	N/A	4.805976226	0.020656198
YNL279W	PRM1	4.844370987	1.95E-08
YKL178C	STE3	4.893102557	3.94E-06
YGR256W	GND2	5.020031299	1.13E-05
YLR213C	CRR1	5.107021716	1.34E-08
YJL045W	N/A	5.135315465	0.000160044
YGR225W	AMA1	5.208836344	5.76E-05
YOR381W-A	N/A	5.226484213	0.012957067
YBR300C	N/A	5.299015014	0.040212393
YJR159W	SOR1	5.375812333	0.044190722
YBL109W	N/A	5.414210992	0.037466018
YFL012W-A	N/A	5.443009987	0.032783019
YKR015C	N/A	5.584766169	1.28E-09
YHR216W	IMD2	5.697250368	8.34E-10
YGL230C	N/A	5.807037567	9.89E-05
YNL204C	SPS18	5.831341694	1.34E-05
YER187W	N/A	5.966596528	6.63E-10
YOL164W-A	N/A	6.074667936	0.012623161
YEL059W	HHY1	6.253861681	0.002061866
YFL040W	N/A	6.423061928	2.16E-10
YGL259W	YPS5	6.777882306	0.00182424
YOL160W	N/A	7.110733586	0.003594576
YOR011W-A	N/A	7.257346649	0.001482249
YFL058W	THI5	7.526137266	0.008858653
YOL091W	SPO21	8.251871931	1.59E-11
YPL272C	PBI1	8.319300732	1.33E-12
YHR184W	SSP1	8.862483068	5.48E-10
YOL047C	LDS2	9.058792333	3.44E-14
YAR035C-A	N/A	9.138880966	0.017808906
YJR025C	BNA1	9.547365631	1.36E-14
YNL332W	THI12	9.595825014	0.002690313
YMR082C	N/A	9.676462199	0.029279763
tV(CAC)H	N/A	9.676462199	0.029279763
YGR260W	TNA1	10.63306735	3.92E-26
YHR015W	MIP6	10.74554343	1.06E-12
YBR148W	YSW1	11.17330536	1.42E-15
YLR307C-A	N/A	11.35640355	0.000131361

YMR232W	FUS2	11.61981836	4.00E-13
RDN5-1	N/A	12.31762217	1.76E-07
YAL066W	N/A	12.9019496	0.002044379
YOR190W	SPR1	13.07724783	3.68E-18
YBL098W	BNA4	13.1633326	3.37E-18
YFL062W	COS4	13.5291277	8.33E-05
YOR298W	MUM3	13.78413419	1.24E-18
YMR321C	N/A	14.01343512	2.41E-06
YJL037W	IRC18	14.11566393	1.35E-10
YOR339C	UBC11	14.8707536	1.06E-09
YPL283C	YRF1-7	15.32106515	0.025463513
YHR185C	PFS1	15.59618025	2.29E-16
YDR542W	PAU10	16.127437	0.001984495
YGR273C	N/A	17.43431551	6.93E-07
YJL038C	LOH1	17.50108207	2.28E-18
YDR042C	N/A	17.65173992	1.23E-17
YCL048W-A	N/A	17.84914987	8.22E-08
YPR078C	N/A	17.98895499	1.54E-13
YOR313C	SPS4	18.46788212	1.44E-20
YJL043W	N/A	18.88178579	9.01E-14
YFR023W	PES4	18.90215753	3.92E-19
YHR125W	N/A	19.11101284	4.31E-05
YLR343W	GAS2	19.73629477	1.13E-28
YAR075W	N/A	21.23445871	0.000595402
YOL024W	N/A	21.40550729	1.22E-05
YLR461W	PAU4	23.78796957	0.000171501
YBR180W	DTR1	24.96425472	3.56E-25
YER084W-A	N/A	24.99752735	0.00466704
YKL177W	N/A	25.11272333	2.06E-05
YAR073W	IMD1	26.87906166	4.09E-06
YBR040W	FIG1	27.7293177	5.96E-14
YGR059W	SPR3	27.75348177	1.33E-25
YFL012W	N/A	28.07640168	3.66E-13
YER106W	MAM1	29.48791177	2.98E-15
YPL027W	SMA1	32.67299273	2.86E-11
YGL170C	SPO74	33.27469722	1.39E-20
YJR078W	BNA2	33.5208778	3.01E-28
YMR244W	N/A	33.5549429	6.43E-17
YDR218C	SPR28	33.66912617	2.88E-20
YDR522C	SPS2	37.33958718	9.09E-37
YCL026C-A	FRM2	39.98260423	2.20E-16

YPL021W	ECM23	42.73770805	0.000498305
YHR126C	ANS1	44.69603968	2.31E-10
YFL011W	HXT10	45.01570852	1.00E-19
YBR045C	GIP1	45.60161946	2.84E-34
YGR259C	N/A	46.56797433	1.94E-06
YOL132W	GAS4	46.76341179	4.71E-32
YDL114W	N/A	61.2842606	3.96E-17
YPL033C	SRL4	62.26593067	1.67E-18
YGL015C	N/A	62.59461485	3.81E-19
YML047C	PRM6	67.48712098	9.81E-14
YOR214C	SPR2	81.72815867	2.42E-19
YPL130W	SPO19	83.32509116	9.63E-11
YDR523C	SPS1	90.81538968	1.10E-36
YBR200W-A	N/A	94.74869237	1.32E-13
YGL262W	N/A	102.4092249	1.27E-07
YOL165C	AAD15	124.9876367	1.78E-06
YFR032C	RRT5	126.9171694	1.60E-37
RUF22	N/A	130.6322397	1.14E-08
YLR307W	CDA1	132.814187	9.20E-43
YKR034W	DAL80	150.1464385	1.21E-14
YGL263W	COS12	158.7311972	6.90E-29
YPL187W	MF(ALPHA)1	165.6163723	6.64E-30
YCL048W	SPS22	192.0099927	1.54E-51
YOR255W	OSW1	226.8208818	1.78E-25
YAL018C	LDS1	321.3917717	1.23E-50
YLR308W	CDA2	1178.310866	4.41E-55
YNL318C	HXT14	1291.001332	1.23E-61
YGL138C	N/A	1445.287146	4.17E-68

Supplemental Table 1. List of up-regulated genes in *sum1*Δ cells compared to wild-type cells.

Systematic Name	Gene Name	Fold Change	p-value
YLR154W-E	N/A	0.007954346	1.43E-36
YDR310C	SUM1	0.024863945	5.58E-27
YMR290W-A	N/A	0.218677112	0.039145928
YPR053C	N/A	0.277190323	0.048441291
YNR069C	BSC5	0.438410502	0.011028242
LSR1	GTS1	0.511322177	0.018727608
YGR023W	MTL1	0.523739856	0.016743233
YMR053C	STB2	0.529436063	0.024390138
YDL184C	RPL41A	0.549172748	0.004015633
YOL158C	ENB1	0.558785338	0.021999907
YCR097W	HMRA1	0.560888468	0.020191057
YDL039C	PRM7	0.565067503	0.015503649
YGR008C	STF2	0.56750462	0.016803479
YMR066W	SOV1	0.570806828	0.036213918
YBL026W	LSM2	0.574696047	0.03828476
YDL133C-A	RPL41B	0.593553692	0.011568373
YNL160W	YGP1	0.597211541	0.026759813
YNL259C	ATX1	0.59956841	0.03915263
YGL178W	MPT5	0.617350098	0.041686235
YFR032C-A	RPL29	0.617710432	0.018476205
YEL003W	GIM4	0.622356313	0.030466938
snR86	n/a	0.631685494	0.042111057
YOL109W	ZEO1	0.639004023	0.038365457

Supplemental Table 2. List of down-regulated genes in *sum1*Δ cells compared to wild-type cells.

GO:0000003	reproduction	0.21%	1
GO:0022414	reproductive process	0.12%	0.955
GO:0032502	developmental process	1.39%	0.956
GO:0032505	reproduction of a single-celled organism	0.13%	0.666
GO:0051321	meiotic cell cycle	0.02%	0.626
GO:1903046	meiotic cell cycle process	0.02%	0.594
GO:0034293	sexual sporulation	0.00%	0.337
GO:0044703	multi-organism reproductive process	0.07%	0.645
GO:0044702	single organism reproductive process	0.10%	0.615
GO:0019953	sexual reproduction	0.05%	0.553
GO:0003006	developmental process involved in reproduction	0.08%	0.383
GO:0022413	reproductive process in single-celled organism	0.00%	0.62
GO:0051704	multi-organism process	2.77%	0.956
GO:0071554	cell wall organization or biogenesis	1.11%	0.927
GO:0071852	fungal-type cell wall organization or biogenesis	0.00%	0.767
GO:0045229	external encapsulating structure organization	0.90%	0.729
GO:0007049	cell cycle	1.41%	0.873
GO:0043934	sporulation	0.08%	0.544
GO:0009435	NAD biosynthetic process	0.21%	0.824
GO:0034354	'de novo' NAD biosynthetic process from tryptophan	0.01%	0.841
GO:0034627	'de novo' NAD biosynthetic process	0.01%	0.841
GO:0022402	cell cycle process	0.53%	0.747
GO:0070726	cell wall assembly	0.00%	0.72
GO:0009272	fungal-type cell wall biogenesis	0.00%	0.653
GO:0071940	fungal-type cell wall assembly	0.00%	0.634
GO:0031505	fungal-type cell wall organization	0.00%	0.644
GO:0042244	spore wall assembly	0.00%	0.405
GO:0030476	ascospore wall assembly	0.00%	0.211
GO:0070591	ascospore wall biogenesis	0.00%	0.211
GO:0042546	cell wall biogenesis	0.80%	0.648
GO:0071555	cell wall organization	0.79%	0.639
GO:0070590	spore wall biogenesis	0.00%	0.446
GO:0048646	anatomical structure formation involved in morphogenesis	0.13%	0.528
GO:0030154	cell differentiation	0.28%	0.485
GO:0044767	single-organism developmental process	1.35%	0.466
GO:0048468	cell development	0.13%	0.466

GO:0010927	cellular component assembly involved in morphogenesis	0.02%	0.418
GO:0009653	anatomical structure morphogenesis	1.01%	0.481
GO:0048856	anatomical structure development	1.24%	0.481
GO:0030435	sporulation resulting in formation of a cellular spore	0.05%	0.451
GO:0048869	cellular developmental process	1.06%	0.456
GO:0032989	cellular component morphogenesis	0.85%	0.358
GO:0000755	cytogamy	0.00%	0.576
GO:0030437	ascospore formation	0.00%	0.314
GO:0043935	sexual sporulation resulting in formation of a cellular spore	0.00%	0.318

Supplemental Table 3. List of upregulated genes grouped into GO terms. All genes listed in Supplemental Table 1 were further grouped into GO terms using the *Saccharomyces* Genome Database GO Annotation algorithm. Frequency and uniqueness of each GO term were calculated during further grouping of GO terms using REVIGO. Frequency describes how general a GO term is, where a higher frequency represents a more general GO term. Uniqueness describes the similarity of the GO term to other GO terms where a higher value represents less similarity.

Strains	Genotype	Source
BY4741	*MATa his3Δ1 leu2Δ0 met15Δ0 ura3Δ0*	Open Biosystems
RHY009	*MATa his3Δ1 leu2Δ0 met15Δ0 ura3Δ0 sir2Δ*::*LEU2*	[8]
RHY010	*MATa his3Δ1 leu2Δ0 met15Δ0 ura3Δ0 sir2Δ*::*LEU2* [pmito-roGFP1:*URA3*]	[8]
RHY016	*MATa his3Δ1 leu2Δ0 met15Δ0 ura3Δ0 SIR2-3HA-kanMX6 sum1Δ::LEU2*	This study
RHY018	*MATa his3Δ1 leu2Δ0 met15Δ0 ura3Δ0 SIR2-3HA-kanMX6 sum1Δ::LEU2* [pmito-roGFP1:*URA3*]	This study
RHY025	*MATa his3Δ1 leu2Δ0 met15Δ0 ura3Δ0* [pmito-roGFP1:*URA3*]	[8]
RHY031	*MATa his3Δ1 leu2Δ0 met15Δ0 ura3Δ0 SIR2-3HA-kanMX6* [pmito-roGFP1:*URA3*] [pRS413-*SIR2-3HA:HIS3*]	[8]
RHY036	*MATa his3Δ1 leu2Δ0 met15Δ0 ura3Δ0 SIR2-3HA-kanMX6 sum1Δ::LEU2 sir2Δ::kanMX6* [pmito-roGFP1:*URA3*]	This study
RHY050	*MATa his3Δ1 leu2Δ0 met15Δ0 ura3Δ0 ABP140- GFP(S65T)-KanMX6*	[8]
RHY088	*MATa his3Δ1 leu2Δ0 met15Δ0 ura3Δ0 ABP140- GFP(S65T)-KanMX6 sum1Δ::LEU2*	This study
RHY099	*MATa his3Δ1 leu2Δ0 met15Δ0 ura3Δ0 SIR2-3HA-kanMX6 sum1Δ::LEU2* [pRS413-*SIR2-3HA:HIS3*]	This study
RHY 104	*MATa his3Δ1 leu2Δ0 met15Δ0 ura3Δ0 SIR2-3HA-kanMX6 sum1Δ::LEU2* [pRS413-*SIR2-3HA:HIS3*] [pmito-roGFP1:*URA3*]	This study
RHY117	*MATa his3Δ1 leu2Δ0 met15Δ0 ura3Δ0 SIR2-3HA-kanMX6 sum1Δ::0 sir2Δ::KanMX6*	This study
RHY124	*MATa his3Δ1 leu2ΔTUB1-GFP::LEU2 met15Δ0 ura3Δ0*	This study
RHY126	*MATa his3Δ1 leu2ΔTUB1-GFP::LEU2 met15Δ0 ura3Δ0 sir2Δ::LEU2*	This study
RHY128	*MATa his3Δ1 leu2ΔTUB1-GFP::LEU2 met15Δ0 ura3Δ0 SIR2-3HA-kanMX6 sum1Δ::LEU2*	This study
RHY130	*MATa his3Δ1 leu2ΔTUB1-GFP::LEU2 met15Δ0 ura3Δ0 sum1Δ::0 sir2Δ::KanMX6*	This study

Supplemental Table 4. Strains used in this study.

**Purpose**	**Forward Primer**	**Reverse Primer**	**Forward Sequencing Primer**	**Reverse Sequencing Primer**
*sir2*Δ using *KanMX6*	CCATTCTCAC GTATTTCAAG AAATTAGGCA TCGCTTCGG CGGATCCCC GGGTTAATTA A	GACTACAATA ATTGAAAGG AAAACAAAAT TGTTTGCCG AATTCGAGCT CGTTTAAAC	AATGATTTGA ATTTGCTGTT CCACC	CCAACCATG GTCCAGGAC AGCCAG
*sum1*Δ using *LEU2*	AAGTTTCATA CATAATTAAC AAAATTCGTT TGTTGCGGG GTGCAGGTC GACAACCCT TAAT	TTTTTATCTA TTCTCGAAAC TGCCCCAAC GTACGGACC AGCGCAGCG TACGGATATC ACCTA	CAGCAAACA GAG CACAAGGGA CTT G	GGTTACTTG GTT GGGCATAGT AAC GGTCC

Supplemental Table 5: **List of deletion primers used in this study.**
